# Reasons for non-participation in a parental program concerning underage drinking: a mixed-method study

**DOI:** 10.1186/1471-2458-9-478

**Published:** 2009-12-21

**Authors:** Camilla Pettersson, Margareta Lindén-Boström, Charli Eriksson

**Affiliations:** 1 School of Health and Medical Sciences, Örebro University, S-701 82 Örebro, Sweden; 2 Örebro County Council, Department of Community Medicine, S-701 16 Örebro, Sweden

## Abstract

**Background:**

Alcohol consumption among adolescents is a serious public health concern. Research has shown that prevention programs targeting parents can help prevent underage drinking. The problem is that parental participation in these kinds of interventions is generally low. Therefore, the aim of the present study is to examine non-participation in a parental support program aiming to prevent underage alcohol drinking. The Health Belief Model has been used as a tool for the analysis.

**Methods:**

To understand non-participation in a parental program a quasi-experimental mixed-method design was used. The participants in the study were invited to participate in a parental program targeting parents with children in school years 7-9. A questionnaire was sent home to the parents before the program started. Two follow-up surveys were also carried out. The inclusion criteria for the study were that the parents had answered the questionnaire in school year 7 and either of the questionnaires in the two subsequent school years (n = 455). Multinomial logistic regression analysis was used to examine reasons for non-participation. The final follow-up questionnaire included an opened-ended question about reasons for non-participation. A qualitative content analysis was carried out and the two largest categories were included in the third model of the multinomial logistic regression analysis.

**Results:**

Educational level was the most important socio-demographic factor for predicting non-participation. Parents with a lower level of education were less likely to participate than those who were more educated. Factors associated with adolescents and alcohol did not seem to be of significant importance. Instead, program-related factors predicted non-participation, e.g. parents who did not perceive any need for the intervention and who did not attend the information meeting were more likely to be non-participants. Practical issues, like time demands, also seemed to be important.

**Conclusion:**

To design a parental program that attracts parents independently of educational level seems to be an important challenge for the future as well as program marketing. This is something that must be considered when implementing prevention programs.

## Background

Parents as well as peers play an important role in the development of adolescents' alcohol habits [1]. In a review of individual risk factors for adolescents' substance use, Swadi [2] showed that the influences of peers as well as family and parent issues are important environmental factors. However, the author also concluded that there are many potential risk factors and there is a complexity of interactions between them. In another review, Kumpfer et al [3] assert that parents have been lead to believe that negative peer influences is the primary reason for drug abuse and other unhealthy behaviours among adolescents. Important is that positive parent influences is as important as the influences of peers. A good relationship within the family [4] and that parents' get knowledge about their child's everyday life by child disclosure [5] is protective factors for alcohol consumption and other norm breaking behaviours. There is a growing body of literature about parental and family programs aiming to reduce risk behaviour among young people [e.g. [6-8]]. A meta-analysis of family program targeting general populations showed effects on alcohol consumption among the adolescents, but the authors emphasize the need of more studies and the importance of studies carried out outside of the US [9]. Examples of successful universal programs targeting families' independently of risk levels are Preparing for the Drug Free Years [7] and Iowa Strengthening Families Program [6]. The latter have been translated and adapted into 17 countries [10]. The Swedish government has emphasized the importance of effective parental support programs where parents are encouraged to have a restrictive attitude towards adolescents and alcohol, and to uphold the minimum age for alcohol consumption (18 years old) [11]. Many Swedish parents have a positive attitude towards structured parental support groups, but only 20% reported they would participate if invited [12]. In a review of family-based drug abuse prevention programs it was found that it is a major challenge to increase the recruitment and retention of parents for participation. In a typical parent prevention program, 40-50% of the eligible parents participate, but it is not unusual the participation rates are much lower [13]. In this study we investigate why the majority of parents choose not to participate in a parental support program to prevent underage drinking.

### Factors influencing participation in parental support programs

A wide range of factors influence the decision whether or not to participate in parental support programs. Socio-economic factors such as income and education have been reported to be of importance [14-17] as well as psychological factors, like fear of being judged by others [18] and the perceived need for a program [19]. Practical issues like time demands [19] and program factors [20] could also affect the decision whether to participate. Factors that influence participation in parental programs could roughly be divided into three categories: socio-demographic factors, psychological and behavioural factors, and finally practical and program-related factors.

#### Socio-demographic factors

Differences in socio-demographic background between participating and non-participating parents have been shown in several studies [14-17,21]. One important factor seems to be parents' educational level with highly educated parents participating to a greater degree than parents with a lower educational level [14-17]. Gender is another factor that in some studies has been a predictor of participation. Fathers seem to have lower interest in parental programs than mothers [12,22,23]. Mothers perceive family-focused prevention programs as more beneficial than fathers [24]. Families with girls appear to be more likely to participate than families with boys [14,25]. There are inconsistent results from previous studies of the significance of other socio-demographic factors. Some studies indicate that marital status [14,21] and income [21] are important, and some do not [17]. The conclusion from previous studies is that socio-demographic factors are important for the decision about participation, but there are some inconsistent results between the studies about the importance of a single factor.

#### Psychological and behavioural factors

Psychological factors, like fear of being judged by others, can play an important role when parents consider participation [18], especially when the programs are targeting parents of children with behavioural problems [26]. The child's behaviour could also affect the decision whether to participate in a parental program. In some studies, parents who perceive their children have problems, like externalizing problems [27] and higher levels of antisocial behaviour [17], were more likely to participate than others. Dumas et al also found a positive association between mother's level of personal or family stress and the intention to enrol in a program to promote effective parenting [21]. In a study of barriers to participation, one important reason for non-participation was that parents perceived that they already were parenting effectively [19]. In other studies no association between family risk factors, such as distress in parent-child relationship, and parental participation in a family intervention program have been found [28]. Instead, parents who attended a drug abuse prevention program reported spending more time together with their children than parents not attending the program [29]. The conclusion could be that some interventions reach those in special need and some do not, but it is important to emphasize that some interventions are universal, i.e. target all families independently of risk level.

#### Practical and program-related factors

Factors associated with practical issues have frequently been identified as barriers to participation in parental programs. Time demands seem to be the most common reason for non-participation [19,26,30]. It is not unusual that parental programs implemented in the US offer childcare and transportation or cash to cover transportation for those who need it [e.g. [20,21]]. Some of the practical barriers are easy to overcome if you have economic resources, but some others are not.

The format of a program can also have an influence on parents' choices about participation. In some studies, self-administered parental programs were considered more attractive [18,20] than other programs. It is reasonable to believe that some parents feel uncomfortable about discussing these family matters with people outside the family. However, in a study addressing barriers to participation in a family-skill preventive intervention, the majority of the parents did not mention this reason for declining to participate [19].

There is a need for research on reasons for non-participation in universal parental prevention programs. Many programs have been studies of efficacy, which means that their ecological validity can be questioned [31]. Therefore, studies of parental prevention programs with ordinary resources in different contexts are badly needed.

### Towards a theory-driven analysis of non-participation

In this study the Health Belief Model (HBM) will be used to study reasons for non-participation in a parental program aimed at preventing underage drinking. The model was developed to explain participation in public health programs and it has been used for decades to explain health-related behaviours [32,33]. The HBM and other social cognitive models have been tested on a variety of health behaviours including alcohol use, dietary practice, health screening activities and visits to health professionals. Meta-analysis of these studies suggest that many health behaviour can consistently but on a modest level be predicted by the components of the HBM [34]. There have been several refinements of the model, but some components were included in the model at an early stage [35]. *Perceived susceptibility *and *perceived severity *deals with individual perceptions about a specific condition or problem. If people believe that they are susceptible to a problem, and if they feel that the problem is serious, the chance that they will participate in an intervention will increase. But they will also weigh the *perceived benefits *against the *perceived barriers *before deciding about participation. Another component, *cue to action*, was also discussed in early versions of the model. A cue to action is something that can trigger an action, like media publicity and information campaigns [36].

**Figure 1:**
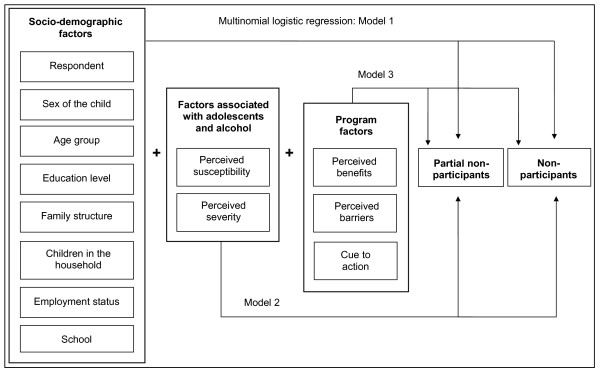
**A HBM inspired theoretical model (for analysis)**.

There has been limited research using the HBM to understand people's decision about participation in parental programs [15,16,37]. Spoth and Redmond have used some of the components of the HBM in their study of parents' inclination to enrol in a parenting skills program [37]. In later studies factors associated with actual participation have been examined [15,16]. It was found that inclination to enrol predicted actual participation and that parents with higher levels of education were more likely to participate than others. As far as we know, no one has used all the components in the model as a tool to understand non-participation in a parental program. The theoretical model used in the present study includes three levels (Figure 1). The first level consists of socio-demographic factors. According to Janz et al socio-demographic factors influence a person's perception of susceptibility, severity, benefits and barriers, and have only an indirect effect on behaviour [36]. The second level contains factors associated with parents' perceptions about adolescents and alcohol. Perceived severity and susceptibility are together called perceived threats, and high levels of threats of this kind have been shown to be a good predictor of intention to engage in behaviour [36]. In the final level factors related to the present program are included. If perceived threats are high, perceived barriers and benefits will be strong predictors of the behaviour sought for. This will also be true for cue to action [36].

The aim of the present study is to examine non-participation in a parental support program to prevent underage alcohol drinking. Three research questions are addressed: (i) Is there any association between socio-demographic factors and non-participation? (ii) Do factors associated with adolescents and alcohol, i.e. perceived susceptibility and perceived severity, matter for non-participation? (iii) How important are program and practical factors such as perceived benefits, perceived barriers and cues to action for non-participation?

## Methods

The present study has a quasi-experimental design, where parents participating in a parental program are compared to non-participating parents. Differences between parents before they were invited to the program are examined as well as parents' own perception of reasons for non-participation after the program was implemented. A mixed-method design is used with quantitative data from a longitudinal survey which follows parents over the three years of secondary school and qualitative data from the last questionnaire in the set of surveys. A mixed-method design is a suitable method when complex issues are addressed [38].

### Participants and Procedure

The participants in this study are parents who have been invited to a parental program called "Strong and Clear" (Stark och klar), which is a universal program aiming to prevent alcohol consumption among adolescents. The program includes thirteen activities during the three years of secondary school. These comprise both group activities and self-administrated activities. The parents could sign up for the program during the whole period it was carried out. The program is implemented by the Swedish temperance organization, IOGT-NTO, which is a part of the global International Organization of Good Templars. Six Swedish schools were chosen for the study. The schools were located in three municipalities in the province of Värmland. The selection of the schools was made in cooperation with IOGT-NTO, and the chosen schools had all been working with the program in previous years.

The study includes an annual survey with questionnaires to parents with adolescents in the six selected schools. Ethical approval was given by the local ethics committee at Örebro University (Dnr CF2003/773) and the regional ethics committee in Uppsala (Dnr 2006-078). The parents were informed by a cover letter describing the study procedure and gave their informed consent by returning the mailed questionnaire.

The baseline surveys were conducted during the school year 2004/2005 when the adolescents were 13 years old (grade seven). There were 895 parents registered in the participating schools' mailing lists in school year seven. Of those, 613 participated in the baseline survey (68.5%). This first data collection was carried out before the parents were introduced to the program. Two follow-up surveys have been made in school years 2005/2006 (grade eight) and 2006/2007 (grade nine). The response rates were 476 of 889 parents (53.5%) and 411 of 901 (45.6%) respectively. In total, 718 parents had answered one or several of the questionnaires. In the two follow-up questionnaires the parents were asked about their participation in the program. Inclusion criteria for the present analysis of non-participation were that the parents answered the questionnaire in school year seven and either of the questionnaires in the two subsequent school years. Parents who only had answered the questionnaire in school year 7 (158 parents) or only in school year eight or nine (105 parents) were therefore excluded from the study. This narrows the analytical sample to 455 parents.

Logistic regression analysis was used to study the attrition in the longitudinal study. A first model examined whether school, sex of the respondent, parental age, family structure, educational level, employment status, number of children in the household, and the child's sex predicted parents leaving the study. The analytical sample, consisting of 455 parents, was compared with those who only answered the questionnaire in school year 7 (n = 158). Parents' educational level was the only significant predictor of attrition. Parents with secondary school as their highest level of education were more likely than parents with university education to only have answered the questionnaire in school year 7 (OR 1.8, CI 1.2-2.8). In the second model, measures of parents' attitudes and behaviour concerning adolescents' alcohol consumption, parents' perceptions about their child's alcohol use, and their interest in participation in a parental support program were added. Parents' educational level was also important in the extended model (OR1.7, CI 1.02-2.7). Number of children in the household and employment status also predicted attrition in this model. Parents with two children in the household were less likely to only have answered the questionnaire in school year 7 than those with one child in the household (OR 0.5 CI 0.3-0.98). Parents outside the labour market were more likely to drop out of the survey than parents working full-time (OR 2.3, CI 1.1-4.8). None of the factors concerning adolescents and alcohol or parents' interest in participation in a parental program were associated with parental attrition. To conclude, parents in the analytical sample had a higher educational level, were more likely to have two children in the household, and were full-time workers to a greater extent than those lost to attrition.

### Measures

#### Participation in Strong and Clear

The parents were classified into three groups according to their responses in the parental follow-up surveys. One group included participants in the program (n = 124). The second group included parents who were partial non-participants. They had attended some activities in Strong and Clear but they did not consider themselves participants in the program (n = 82). The partial non-participants had completed less numerous of activities than the participants, in average 0.6. The third group consisted of non-participating parents who had not attended any activities in Strong and Clear except the first information meeting in school year seven (n = 249).

#### Socio-demographic factors

The socio-demographic factors included in this study are sex of the respondent, sex of the child, parental age, parental education, family structure, number of children in the household, parental employment status, and school (Table 1). Six of these eight factors concern the parents' background. In this study, the sex of the child describes the adolescents' demographic background. The information about the parents' socio-demographic background is taken from the parental survey in school year seven. If the parents have answered the questionnaire together, the highest reported age, the highest educational level, and the highest employment status are used in the analysis.

**Table 1:** Descriptive statistics of the study population.

	Total Per cent (n) ↓	Participants Per cent (n) →	Partial non-participants Per cent (n)	Non-participants Per cent (n)	Total Per cent (n)
**Respondent**	**100 (441)**				
Mother and father together	12.2 (54)	38.9 (21)	13.0 (7)	48.1 (26)	**100 (54)**
Mother	76.0 (335)	27.2 (91)	18.5 (62)	54.3 (182)	**100 (335)**
Father	11.8 (52)	19.2 (10)	15.4 (8)	65.4 (34)	**100 (52)**
					
**Sex of the child**	**100 (448)**				
Girl	47.8 (214)	27.6 (59)	18.2 (39)	54.2 (116)	**100 (214)**
Boy	52.2 (234)	26.5 (62)	18.4 (43)	55.1 (129)	**100 (234)**
					
**Age group**	**100 (449)**				
Age group 1 = quartile 1	23.6 (106)	19.8 (21)	18.9 (20)	61.3 (65)	**100 (106)**
(29-39 years old)					
Age group 2 = quartiles 2 & 3	51.2 (230)	29.1 (67)	19.6 (45)	51.3 (118)	**100 (230)**
(40-46 years old)					
Age group 3 = quartile 4	25.2 (113)	31.9 (36)	13.3 (15)	54.9 (62)	**100 (113)**
(47-67 years old)					
					
**Education level**	**100 (451)**				
Tertiary/university	53.4 (241)	36.5 (88)	17.0 (41)	46.5 (112)	**100 (241)**
Secondary	44.6 (201)	16.9 (34)	18.9 (38)	64.2 (129)	**100 (201)**
Other forms of education	2.0 (9)	22.2 (2)	11.1 (1)	66.7 (6)	**100 (9)**
					
**Family structure**	**100 (452)**				
Household with more than one adult	85.8 (388)	27.1 (105)	17.8 (69)	55.2 (214)	**100 (388)**
Household with one adult	14.2 (64)	29.7 (19)	17.2 (11)	53.1 (34)	**100 (64)**
					
**Children in the household**	**100 (450)**				
One child	10.7 (48)	22.9 (11)	20.8 (10)	56.2 (27)	**100 (48)**
Two children	51.3 (231)	28.6 (66)	16.9 (39)	54.5 (126)	**100 (231)**
Three or more children	38.0 (171)	26.9 (46)	18.1 (31)	55.0 (94)	**100 (171)**
					
**Employment status**	**100 (448)**				
Full-time job	62.7 (281)	29.9 (84)	18.5 (52)	51.6 (145)	**100 (281)**
Part-time job	29.2 (131)	24.4 (32)	15.3 (20)	60.3 (79)	**100 (131)**
Outside labour market	8.0 (36)	19.4 (7)	19.4 (7)	61.1 (22)	**100 (36)**
					
**School**	**100 (455)**				
School 1 (Highest participation rate in Strong & clear)	15.8 (72)	41.7 (30)	12.5 (9)	45.8 (33)	**100 (72)**
School 2	8.1 (37)	32.4 (12)	18.9 (7)	48.6 (18)	**100 (37)**
School 3	16.3 (74)	18.9 (14)	14.9 (11)	66.2 (49)	**100 (74)**
School 4	11.4 (52)	28.8 (15)	13.5 (7)	57.7 (30)	**100 (52)**
School 5	25.7 (117)	23.9 (28)	21.4 (25)	54.7 (64)	**100 (117)**
School 6	22.6 (103)	24.3 (25)	22.3 (23)	53.4 (55)	**100 (103)**

#### The Components of the Health Belief Model

The components of the HBM are measured by 10 questions. In Table 2 the operationalization of the major components and the sources of information are presented. The components of the model are examined by quantitative data with one exception: barriers to participation. Barriers were examined with an open ended question and analysed by content analysis. The two largest categories have been used as dichotomous variables in the quantitative analysis.

**Table 2:** Operationalizing of the Major Components of the HBM, Source of Information and Proportions of Response.

Survey year	Question	Response alternatives		Per cent (n)
**Perceived susceptibility**				

School year 7	Has your son/daughter drunk alcohol outside of the home?	1. Absolutely not		1: 77.0 (348)
		2. Probably not		2, 5: 21.7 (98)
		3. Yes, once		3, 4: 1.3 (6)
		4. Yes, more than once		
		5. I do not know		

School year 7	Are you worried that your son/daughter will drink alcohol outside the home?	1. No, not at all		1, 2: 70.3 (317)
		2. No, not particularly		3: 20.6 (93)
		3. Yes, a little		4, 5: 9.1 (41)
		4. Yes, rather much		
		5. Yes, very much		

**Perceived severity**				

School year 7	When do you think it is okay for adolescents to drink alcohol?	1. -13 years old	5. 17 years old	1, 2, 3, 4, 5: 7.8 (34)
		2. 14 years old	6. 18 years old	6, 7, 8: 92.2 (402)
		3. 15 years old	7. 19 years old -	
		4. 16 years old	8. Never	

School year 7	Has your child ever been offered alcohol at home?	1. No we don't drink alcohol in our family		1, 2: 65.6 (297)
		2. No		3, 4, 5: 34.4 (156)
		3. Yes, he/she has been allowed to take a sip from a glass		
		4. Yes, he/she has been served alcohol in their own glass		
		5. Yes, he/she has often been served alcohol in their own glass		

School year 7	Which of the following statements is closest to your opinion about adolescents and alcohol?	1. Adolescents at my child's age are mature enough to handle alcohol in a responsible way.		1,2: 18.0 (79)
		2. I am not in favour of adolescents in the same age group as my child using alcohol, but I do not think adults can do anything about it.		3: 82.0 (361)
		3. To me it is obvious that adolescents under 18 years should not concern themselves with alcohol		

**Perceived benefits**				

School year 7	Would you like to participate in a parental support group to promote your child's development and health?	1. No		1: 8.5 (38)
		2. Yes		2: 56.3 (253)
		3. I do not know		3: 35.2 (158)

School year 7	What do you think can prevent alcohol use among adolescents?	Prevent: 1. Not at all		1, 2: 25.4 (115)
	- Values shared in common with other parents in the child's class	2. Somewhat		3: 40.8 (185)
		3. Rather much		4: 33.8 (153)
		4. Much		

**Perceived barriers**				

School year 9	Why did you not participate in Strong & Clear?	An open ended question:		Total: 176 statements
		1. Absence of perceived needs^1^		1. 19. 1 (87)
		2. Practical barriers^1^		2. 10. 1 (46)

**Cue to action**				

School year 8-9	Have you been informed about Strong & Clear?	1. Yes, I have received an invitation to the parental meeting at school		1: 63.1 (282)
		2. Yes, I have seen the agreement after a parental meeting		2: 4.0 (18)
		3. No		3: 22.8 (102)
		4. I have received an invitation to the parental meeting at school and seen the agreement after a parental meeting		4: 10.1 (45)

School year 8-9	Were you at the information meeting about Strong & Clear in school year 7?	1. Yes		1: 79.3 (352)
		2. No		2: 20.7 (92)

^1 ^The two largest categories from the content analysis

The operationalization of the components in the HBM was first done theoretically (Figure 1). To control for internal validity of components, two factor analyses were performed. The first factor analysis was done using the five items about adolescents and alcohol that were theoretically defined as perceived susceptibility and perceived severity (Table 2). All parents who had answered any of the questionnaires in the longitudinal survey were used in the factor analysis (n = 718). Principal component analysis was used as the extraction method and oblimin with Kaiser Normalization was used as the rotation method, because correlation between the factors was expected [39]. This analysis revealed the presence of two factors with eigenvalues above 1.0. The two factors explained a total of 57.8% of the variance, with factor one contributing 34.0% and factor two 23.8%. The factor analysis showed that the two items regarding perceived susceptibility measured a different latent factor than the three items regarding perceived severity (Table 3). Moreover, the results did not show a high correlation between the factors (0.169).

**Table 3:** Factor analysis of the items measuring perceived susceptibility and perceived severity.

	Factor 1	Factor 2
	Perceived susceptibility	Perceived severity
Adolescent alcohol use outside home	**0.834**	0.182
Worries about adolescent alcohol use	**0.843**	0.104
Approved age for alcohol use	0.085	**0.657**
Adolescents have been offered alcohol at home	-0.165	**-0.731**
Parents' opinion about adolescents' alcohol use	-0.112	**-0.714**

*Note*. Numbers in boldface are significant factor loadings.

Furthermore, a factor analysis was performed with the program-related items defined in the theoretical model as perceived benefits and cues to action. The item measuring perceived barriers was not included in the factor analysis, as it was an open-ended question. This analysis revealed the presence of two factors with eigenvalues above 1.0. The two factors explained a total of 56.2% of the variance, with factor one contributing 30.4% and factor two 25.8%. The two items operationalized as perceived benefits were revealed in one factor and the two items operationalized as cues to action were revealed as another factor (Table 4). One of the four items loaded on both factors. In subsequent analyses it was used only for perceived benefits. The correlation between the factors was 0.029.

**Table 4:** Factor analysis of the items measuring perceived benefits and cue to action.

	Factor 1	Factor 2
	Perceived benefits	Cue to action
Interest in parental support groups	**0.850**	-0.209
Beliefs in values shared in common with other parents as preventing alcohol use	**0.551**	**0.510**
Received information about Strong & Clear	0.023	**-0.667**
Been at information meeting	-0.069	**-0.683**

*Note*. Numbers in boldface are significant factor loadings.

### Analysis

As a mixed-method design was used, the data were analysed both statistically and with content analysis. In the analysis of the importance of different components of the HBM a more descriptive approach was combined with multivariate analysis. The two groups of non-participants are compared with the group of participating parents. In the analysis of the quantitative data crude odds ratios were calculated. Thereafter, a multinomial regression analysis was applied. Three models were used to analyse the three research questions (Figure 1). In the first model, the importance of socio-demographic factors was calculated (χ^2 ^= 57.48, df = 34, p-value .007). In model 2, the factors associated with adolescents and alcohol were added (χ^2 ^= 70.48, df = 48, p-value .019), and in the last model, program-related factors were also included (χ^2 ^= 194.86, df = 68, p-value p < .001).

The qualitative analysis of perceived barriers was performed by using content analysis on the responses to the opened-ended question in the questionnaires [40]. There were 176 responses, all considered meaning units. The meaning units were then condensed into shorter sentences without changing the core of the statements. The condensed meaning units were tagged with one or several codes. Sub-categories were inductively created based on those codes and classified into eight main categories. The main categories were divided into two main themes. The analysis was carried out by the first author, but before any final decision was made the co-authors read through all the steps in the analysis in order to validate the results.

## Results

### Socio-demographic factors

The crude odds ratios are given in Table 5. Non-participation was more prevalent among fathers than among parents answering the questionnaire together (OR 2.7, CI 1.1-6.8). Parents with secondary education were more likely to be non-participants than parents with higher educational levels (OR 3.0, CI 1.9-4.8). Less educated parents were also more likely to be partial non-participants, (i.e., taken part in activities in the program but do not consider themselves participants) than parents with better education (OR 2.4, CI 1.3-4.3). Model 1, a multinominal logistic regression including all eight socio-demographic factors, showed that these three odds ratios remained significant after controlling for the other factors (Table 6 and Table 7). There were also some differences between the schools.

**Table 5:** Crude odds ratios (OR) for being a partial non-participant or non-participant in a parental program.

	Partial non-participants	Non-participants
	Crude OR (CI 95%)	Crude OR (CI 95%)
**Socio-demographic factors**		

Respondent		
Mother and father together	Ref.	Ref.
Mother	2.0 (0.8-5.1)	1.6 (0.9-3.0)
Father	2.4 (0.7-8.5)	**2.7 (1.1-6.8)**

Sex of the child		
Girl	Ref.	Ref.
Boy	1.0 (0.6-1.8)	1.1 (0.7-1.6)

Age group		
Age group 3 (47-67 years old)	Ref.	Ref.
Age group 2 (40-46 years old)	1.6 (0.8-3.3)	1.0 (0.6-1.7)
Age group 1 (29-39 years old)	2.3 (1.0-5.4)	1.8 (0.9-3.4)

Education level		
Tertiary/University	Ref.	Ref.
Secondary	**2.4 (1.3-4.3)**	**3.0 (1.9-4.8)**
Other forms of education	1.1 (0.1-12.2)	2.4 (0.5-12.0)

Family structure		
Household with more than one adult	Ref.	Ref.
Household with one adult	0.9 (0.4-2.0)	0.9 (0.5-1.6)

Children in the household		
One child	Ref.	Ref.
Two children	0.7 (0.3-1.7)	0.8 (0.4-1.7)
Three or more children	0.7 (0.3-2.0)	0.8 (0.4-1.8)

Employment status		
Full-time job	Ref.	Ref.
Part-time job	1.0 (0.5-1.9)	1.4 (0.9-2.3)
Outside labour market	1.6 (0.5-4.9)	1.8 (0.7-4.4)

School		
School 1	Ref.	Ref.
School 2	1.9 (0.6-6.4)	1.4 (0.6-3.3)
School 3	2.6 (0.9-7.8)	**3.2 (1.5-6.9)**
School 4	1.6 (0.5-5.0)	1.8 (0.8-4.0)
School 5	**3.0 (1.2-7.5)**	**2.1 (1.1-4.0)**
School 6	**3.1 (1.2-7.8)**	**2.0 (1.0-4.0)**

**Perceived susceptibility**		

Child's alcohol use outside of home		
Absolutely not	Ref.	Ref.
Probably not	1.1 (0.5-.2.1)	1.2 (0.7-2.0)
Yes	3.1 (0.3-35.2)	1.5 (0.2-15.0)

Worries about child's alcohol use		
No	Ref.	Ref.
Yes, a little	0.5 (0.2-1.1)	0.8 (0.5-1.3)
Yes, much	2.1 (0.8-5.5)	1.2 (0.5-2.9)

**Perceived severity**		

Approved age for alcohol use		
18 years old or older	Ref.	Ref.
Under 18 years old	1.9 (0.6-5.9)	1.8 (0.7-4.7)

The child has been offered alcohol at home		
No	Ref.	Ref.
Yes	0.9 (0.5-1.7)	1.0 (0.7-1.6)

Parent's opinion about adolescents' alcohol use		
Restrictive	Ref.	Ref.
Permissive	0.8 (0.3-1.7)	1.1 (0.6-1.9)

**Perceived benefits**		

Interest in parental support groups		
I do not know	Ref.	Ref.
No	1.6 (0.5-5.4)	1.9 (0.8-4.6)
Yes	1.4 (0.8-2.6)	1.0 (0.6-1.6)

Values shared in common with other parents		
Prevent much	Ref.	Ref.
Prevent rather much	0.8 (0.4-1.5)	1.5 (0.9-2.5)
Prevent somewhat or not at all	1.5 (0.7-3.1)	2.1 (1.2-3.9)

**Perceived barriers**		

Absence of perceived needs		
Did not report this as a reason for non-participation	Ref.	Ref.
Reported this as a reason for non-participation	**5.9 (2.6-13.5)**	**3.4 (1.6-7.1)**

Practical barriers		
Did not report this as a reason for non-participation	Ref.	Ref.
Reported this as a reason for non-participation	**11.0 (3.6-33.4)**	2.6 (0.9-7.8)

**Cue to action**		

Information about Strong & Clear		
Yes, received invitation and agreement	Ref.	Ref.
Yes, received invitation	0.9 (0.4-2.0)	1.6 (0.8-3.3)
Yes, received agreement	0.5 (0.1-2.8)	1.8 (0.5-6.0)
No	1.3 (0.4-4.0)	**7.8 (3.1-19.8)**

Been at information meeting		
Yes	Ref.	
No	**3.0 (1.6-5.7)**	**11.1 (6.0-20.3)**

*Note*. Numbers in boldface are significant p < .05.

**Table 6:** Odds ratios for being a partial non-participant, a multinomial logistic regression.

		Partial non-participants		
		**Model 1**	**Model 2**	**Model 3**
		**OR (CI 95%)**	**OR (CI 95%)**	**OR (CI 95%)**

**Socio-demographic factors**				

**Respondent:**	Mother and father together	Ref.	Ref.	Ref.
	Mother	1.9 (0.7-5.3)	2.0 (0.7-5.9)	1.6 (0.5-5.5)
	Father	2.4 (0.6-9.3)	2.8 (0.6-13.0)	3.6 (0.6-20.5)

**Sex of the child:**	Girl	Ref.	Ref.	Ref.
	Boy	1.0 (0.5-1.9)	0.9 (0.5-1.8)	1.1 (0.5-2.2)

**Age group:**	Age group 3 (47-67 years old)	Ref.	Ref.	Ref.
	Age group 2 (40-46 years old)	1.6 (0.7-3.5)	1.6 (0.7-3.7)	1.7 (0.6-4.6)
	Age group 1 (29-39 years old)	2.2 (0.8-5.6)	2.1 (0.8-5.9)	2.3 (0.7-7.5)

**Education level:**	Tertiary/University	Ref.	Ref.	Ref.
	Secondary	**3.3 (1.7-6.3)**	**3.3 (1.6-6.7)**	**3.0 (1.3-6.9)**
	Other forms of education	0.8 (0.1-10.5)	0.7 (0.1-8.8)	0.2 (0.01-5.1)

**Family structure:**	Household with more than one adult	Ref.	Ref.	Ref.
	Household with one adult	0.7 (0.3-1.7)	0.6 (0.2-1.8)	0.7 (0.2-2.2)

**Children in the household:**	One child	Ref.	Ref.	Ref.
	Two children	0.6 (0.2-1.7)	0.7 (0.2-2.0)	0.6 (0.2-2.3)
	Three or more children	0.7 (0.2-1.9)	0.8 (0.3-2.5)	0.5 (0.1-2.1)

**Employment status:**	Full-time job	Ref.	Ref.	Ref.
	Part-time job	0.9 (0.4-1.8)	0.8 (0.4-1.8)	0.8 (0.3-2.0)
	Outside labour market	1.8 (0.5-5.9)	2.4 (0.6-9.8)	4.1 (0.8-20.9)

**School:**	School 1	Ref.	Ref.	Ref.
	School 2	2.0 (0.6-7.3)	1.9 (0.5-7.4)	2.5 (0.5-12.8)
	School 3	**3.7 (1.2-11.8)**	3.1 (0.9-10.8)	**4.6 (1.1-19.8)**
	School 4	1.1 (0.3-4.0)	1.1 (0.3-4.3)	1.0 (0.2-4.6)
	School 5	**4.7 (1.7-12.8)**	**3.9 (1.3-12.1)**	**5.4 (1.5-19.9)**
	School 6	**3.9 (1.4-10.6)**	**3.2 (1.1-9.6)**	**3.8 (1.1-13.8)**

**Perceived susceptibility**				

**Child's alcohol use outside of home:**	Absolutely not		Ref.	Ref.
	Probably not		1.0 (0.4-2.6)	1.6 (0.5-4.5)
	Yes		1.9 (0.1-24.2)	1.5 (0.1-32.9)

**Worries about child's alcohol use:**	No		Ref.	Ref.
	Yes, a little		**0.3 (0.1-0.8)**	**0.2 (0.1-0.6)**
	Yes, much		1.5 (0.5-4.7)	2.1 (0.6-7.6)

**Perceived severity**				

**Approved age for alcohol use:**	18 years old or older		Ref.	Ref.
	Under 18 years old		3.0 (0.7-12.1)	1.0 (0.2-5.0)

**The child has been offered alcohol at home:**	No		Ref.	Ref.
	Yes		1.0 (0.5-2.0)	0.9 (0.4-2.0)

**Parent's opinion about adolescents alcohol use:**	Restrictive		Ref.	Ref.
	Permissive		0.4 (0.1-1.2)	0.3 (0.1-1.1)

**Perceived benefits**				

**Interest in parental support groups:**	I do not know			Ref.
	No			2.9 (0.6-13.9)
	Yes			**3.5 (1.4-8.5)**

**Values shared in common with other parents:**	Prevent much			Ref.
	Prevent rather much			0.7 (0.3-1.8)
	Prevent somewhat or not at all			1.6 (0.6-4.6)

**Perceived barrier**				

**Absence of perceived needs:**	Did not report this as a reason for non-participation			Ref.
	Reported this as a reason for non- participation			**6.2 (2.1-18.0)**

**Practical barriers:**	Did not report this as a reason for non-participation			**Ref**.
	Reported this as a reason for non-participation			**24.7 (5.7-106.3)**

**Cue to action**				

**Information about Strong & Clear:**	Yes, received invitation and agreement			Ref.
	Yes, received invitation			1.2 (0.4-3.9)
	Yes, received agreement			1.3 (0.2-10.7)
	No			1.8 (0.4-9.3)

**Been at Information meeting:**	Yes			Ref.
	No			**2.9 (1.1-7.4)**

*Note*. Numbers in boldface are significant p < .05.

**Table 7:** Odds ratios for being a non-participant, a multinomial logistic regression.

		Non-participants		
		**Model 1**	**Model 2**	**Model 3**
		**OR (CI 95%)**	**OR (CI 95%)**	**OR (CI 95%)**

**Socio-demographic factors**				

**Respondent:**	Mother and father together	Ref.	Ref.	Ref.
	Mother	1.4 (0.7-2.9)	1.3 (0.6-2.7)	1.0 (0.4-2.7)
	Father	**2.8 (1.0-7.7)**	2.9 (1.0-8.8)	2.7 (0.7-10.1)

**Sex of the child:**	Girl	Ref.	Ref.	Ref.
	Boy	0.9 (0.6-1.5)	0.9 (0.6-1.6)	0.9 (0.5-1.6)

**Age group:**	Age group 3 (47-67 years old)	Ref.	Ref.	Ref.
	Age group 2 (40-46 years old)	0.9 (0.5-1.6)	0.9 (0.5-1.6)	0.9 (0.4-1.8)
	Age group 1 (29-39 years old)	1.4 (0.7-2.9)	1.2 (0.6-2.6)	1.0 (0.4-2.5)

**Education level:**	Tertiary/University	Ref.	Ref.	Ref.
	Secondary	**3.3 (2.0-5.6)**	**3.6 (2.1-6.3)**	**2.6 (1.3-5.0)**
	Other forms of education	1.7 (0.3-9.6)	1.9 (0.3-10.8)	1.6 (0.2-12.2)

**Family structure:**	Household with more than one adult	Ref.	Ref.	Ref.
	Household with one adult	0.7 (0.4-1.4)	0.9 (0.4-1.9)	0.7 (0.3-1.7)

**Children in the household:**	One child	Ref.	Ref.	Ref.
	Two children	0.8 (0.3-1.8)	0.7 (0.3-1.8)	0.7 (0.2-2.1)
	Three or more children	0.8 (0.3-1.8)	0.8 (0.3-1.9)	0.5 (0.2-1.6)

**Employment status:**	Full-time job	Ref.	Ref.	Ref.
	Part-time job	1.5 (0.8-2.6)	1.4 (0.8-2.6)	1.5 (0.7-3.1)
	Outside labour market	2.0 (0.7-5.3)	2.8 (0.9-8.7)	3.4 (0.9-13.3)

**School:**	School 1	Ref.	Ref.	Ref.
	School 2	1.8 (0.7-4.7)	1.4 (0.5-3.8)	2.4 (0.7-8.3)
	School 3	**4.3 (1.8-10.2)**	**3.7 (1.5-9.1)**	**5.9 (1.9-18.3)**
	School 4	1.6 (0.7-3.8)	1.3 (0.5-3.3)	1.2 (0.4-3.7)
	School 5	**3.3 (1.5-7.0)**	**2.9 (1.3-6.7)**	**4.4 (1.6-12.0)**
	School 6	**2.9 (1.4-6.2)**	**2.5 (1.1-5.4)**	**3.1 (1.2-8.3)**

**Perceived susceptibility**				

**Child's alcohol use outside of home:**	Absolutely not		Ref.	Ref.
	Probably not		1.3 (0.7-2.5)	1.8 (0.8-3.9)
	Yes		0.8 (0.1-10.0)	0.3 (0.01-8.1)

**Worries about child's alcohol use:**	No		Ref.	Ref.
	Yes, a little		0.6 (0.3-1.1)	0.7 (0.3-1.4)
	Yes, much		0.7 (0.3-1.8)	0.7 (0.2-2.5)

**Perceived severity**				

**Approved age for alcohol use:**	18 years old or older		Ref.	Ref.
	Under 18 years old		1.6 (0.5-5.0)	0.9 (0.3-3.6)

**The child has been offered alcohol at home:**	No		Ref.	Ref.
	Yes		0.9 (0.5-1.5)	0.8 (0.4-1.6)

**Parent's opinion about adolescents alcohol use:**	Restrictive		Ref.	Ref.
	Permissive		0.9 (0.5-1.9)	0.6 (0.2-1.3)

**Perceived benefits**				

**Interest in parental support groups:**	I do not know			Ref.
	No			2.0 (0.6-6.7)
	Yes			1.9 (1.0-3.6)

**Values shared in common with other parents:**	Prevent much			Ref.
	Prevent rather much			1.1 (0.6-2.4)
	Prevent somewhat or not at all			1.9 (0.8-4.4)

**Perceived barrier**				

**Absence of perceived needs:**	Did not report this as a reason for non-participation			Ref.
	Reported this as a reason for non-Participation			**3.4 (1.3-8.7)**

**Practical barriers:**	Did not report this as a reason for non-participation			Ref.
	Reported this as a reason for non-participation			3.8 (0.9-15.3)

**Cue to action**				

**Information about Strong & Clear:**	Yes, received invitation and agreement			Ref.
	Yes, received invitation			1.8 (0.7-4.8)
	Yes, received agreement			2.8 (0.6-12.6)
	No			**8.9 (2.5-31.5)**

**Been at information meeting:**	Yes			Ref.
	No			**7.1 (3.2-15.6)**

*Note*. Numbers in boldface are significant p < .05.

### Factors associated with adolescents and alcohol: Perceived susceptibility and perceived severity

The crude odds ratios for non-participation were not significant for any of the items used to measure the perceived susceptibility or perceived severity (Table 5). Including this block of factors in the multinominal analysis resulted in Model 2 (Table 6 and Table 7). The importance of educational level (OR 3.3 CI 1.6-6.7 and OR 3.6 CI 2.1-6.3 respectively) and school remained significant for both groups of non-participants. Moreover, parents who were somewhat worried about their child's alcohol use were less likely to be partial non-participants than parents who were not worried (0.3 CI 0.1-0.8).

### Program factors: Perceived benefits, perceived barriers and cues to action

The crude odds ratios for the program factors are presented in Table 5. Perceived benefits of the program were non-significant for both groups of non-participants. However, perceived barriers, measured by the two largest categories from the content analysis, had significant crude odds ratios for both groups of non-participant. Parents who had stated that they did not perceive any need for the program were more likely to be partial non-participants (OR 5.9, CI 2.6-13.5) and to be non-participants (OR 3.4, CI 1.6-7.1) than other parents. Moreover, parents who stated that practical barriers were a reason for non-participation were more likely to be partial non-participants (OR 11.0, CI 3.6-33.4) than other parents. Cues to action were significant predictors for both levels of non-participation. Not having attended the information meeting had high odds ratios for both groups of non-participants (OR 3.0, CI 1.6-5.7 and OR 11.1, CI 6.0-20.3 respectively). Not having received any information about Strong and Clear was significant for non-participation (OR 7.8, CI 3.1-19.8).

In Model 3 (Table 6 and Table 7), where all factors were included in the analysis, the importance of educational level (OR 3.0, CI 1.3-6.9 and OR 2.6, CI 1.3-5.0 respectively) and school remained the only significant socio-demographic factors. As in Model 2 the only item related to adolescents and alcohol that became significant was parents' worries about their child's alcohol use. Parents who were somewhat worried about their child's alcohol use were less likely to be partial non-participants than parents who were not worried (0.2 CI 0.1-0.6). When the program factors were included in the multinominal analysis one of the items measuring perceived benefits became significant. Parents who were interesting in participating in a parental support group were more likely to be partial non-participants (OR 3.5, CI 1.4-8.5) than other parents. Perceived barriers seem to be important to the decision whether or not to participate. Absence of a perceived need for the intervention was a significant predictor for both groups of non-participants (OR 6.2, CI 2.1-18.0 and OR 3.4, CI 1.3-8.7 respectively). Parents who had reported practical barriers to participation were more likely to be partial non-participants (OR 24.7, CI 5.7-106.3) than other parents. However, parents who reported this were not more likely to be in the group of non-participants than others. The last factor included in the model was cue to action. Parents who had not received any information about the program were more likely to be non-participants (OR 8.9, CI 2.5-31.5) than those who had been informed. Finally, parents who did not go to the information meeting were more likely to be in one of the groups of non-participants (OR 2.9, CI 1.1-7.4 and OR 7.1, CI 3.2-15.6) than other parents.

To conclude, educational level seems to be the most important socio-demographic factor for predicting non-participation. Parents with lower levels of education were less likely to participate than parents with a higher educational level. There were also some differences between schools that remained significant through all three models. Perceived susceptibility and perceived severity did not seem to be important to the decision whether or not to participate. Instead, program factors seemed to predict non-participation. For example, parents who perceived barriers to participation were more likely to be in one of the groups of non-participants as well as parents who had not been at the information meeting.

### Reasons for non-participation: Perceived barriers

The reasons parents gave for non-participation have been abstracted into two main themes: family-related factors and program-related factors. **Family-related factors **included the following main categories: no perceived need for the intervention, practical barriers, previous experiences, other protective activities, the child opposing participation, and motivational factors.

The category including the most statements was the one concerning parents' perception of the program not being needed. Examples of statements are that the child is not at risk, that the child is already behaving properly, and that the parents are not worried about their child. Some parents stated that they trust their child, and some mentioned that their child was not interested in alcohol, tobacco, or other drugs.

He has a strong opinion about tobacco, alcohol, and other drugs.

Other reasons for non-participation were that the parents always know where their child is or that the child often or always is at home. Some parents claimed that their child never attends parties.

Other reasons related to perceived needs for the intervention were that the family already has a good relationship or good communication.

We communicate without help from consultants.

Good relationship with the child. He knows that he can talk about everything.

We have a good and close relationship.

Some parents stated that they can handle the situation without the program.

We manage it without higher state interference.

We are doing fine as it is.

That the family did many things together and had a strong social network are other examples of the lack of perceived need for the intervention.

Good relationship with son's friends and their parents.

Many statements were related to practical barriers such as issues of time and family situation. There are two different types of time demands: lack of time, and activities being carried out in the evenings, when the parents were prevented from coming.

Working too much.

Evening work, irregular working hours, and travel.

Some parents stated that aspects of their family situation, like single parenthood and illness, were barriers to participation. Others mentioned transportation difficulties.

Previous experiences of alcohol interventions were mentioned by some as reasons for non-participation. These parents had previously participated in the current program, participated in similar programs or received information about drugs. This category included parents who mentioned older children as a reason for non-participation.

We are calm, seventh child.

Some parents reported having a form of employment that gave them enough experience.

Have worked with care of addicts.

Am involved in these issues trough my work within the school health service.

Many parents stated that their child or the whole family was engaged in other protective activities like sports, scouting, or temperance movements.

Another reason for non-participation was that the children opposed it.

Our son felt like we were doubting him and wanted us to show that we trust him.

The final category included in the theme about family-related factors includes statements that indicate poor motivation for participation.

Not important in our family.

Have not prioritized Strong and Clear.

The second main theme was **program-related factors**. It includes statements about the format of the program.

Do not believe in this kind of admonishment.

The program is designed for younger children.

Sports would be a better platform.

Should be part of the regular parents meetings.

Other program-related factors were poor leadership and poor administration. Some parents did not even know about the program. Others have used some of the materials that had been sent home and some stated that they already were doing similar activities.

Used the information for talking with the child.

Includes the components of Strong and Clear in a natural way.

Some parents, despite their non-participation, had a positive attitude toward the program and some thought that the program did not reach those most in need of it.

Some parents had comments about the organization that implemented the program. One parent did not perceive the Swedish temperance organisation (IOGT-NTO) as an attractive organization whereas other parents reported family membership in the organization as a reason for non-participation.

In summary, the most important reason for non-participation was the absence of a perceived need for the program. Time demands also seem to be an important barrier.

## Discussion

The results of the present study indicate that both socio-demographic factors and program- related factors were important for non-participation in a parental program to prevent underage drinking. A finding of equal importance is that factors associated with adolescents and alcohol did not seem to predict non-participation.

### Socio-demographic factors

Of the eight socio-demographic factors included in the analysis, educational level of the parents seemed to be the most important. Parents with a lower level of education were less likely to participate in the program than parents with a higher educational level. This corresponds to previous studies about participation in parental programs [14-17]. To design a parental program that attracts parents with low as well as high educational levels seem to be a very important challenge for the future.

There were some differences between schools that remained significant through all three models. One important point is that the school with the highest rate of participants was used as the reference in the logistic regression analysis which increases the possibility to detect differences. One plausible explanation of the differences could be that the implementation of the program differed between the schools. The actual support from the school director seems to be related to the rate of participation in the program. Implementation of a prevention program depends on many factors, and a recent review article of implementation factors showed that contextual factors are very important for the outcome of an intervention. Moreover, different factors are important at different parts of the implementation process. Community level factor could be related to politics and funding while factors related to the prevention delivery system deals with training to prepare providers effectively in intervention skills [41]. To understand the differences between schools that appeared in this study a deeper analysis of the implementation process and contextual factors is needed.

### Factors associated with adolescents and alcohol: Perceived susceptibility and perceived severity

It is commonly held that prevention programs only reach those who already are convinced. The present study did not confirm this reasoning. There were no differences between the parents' perception about adolescents and alcohol before they were invited to the program. In some studies, it has even appeared that parents perceiving child problems were more likely to participate in parental support programs than others [17,27]. It is important to emphasize that the parental program in the present study is a universal program targeting all parents independently of risk. According to the HBM the target group needs to believe that they can be susceptible to the specific problem that the intervention is meant to prevent [36]. In general, in the present study most parents did not believe that their child was at likely to use alcohol. Only 1.3% of the parents reported that their child had drunk alcohol outside the home and 77.0% of the parents reported that their child had absolutely never consumed alcohol. Moreover, only one out of ten were worried that their child would drink alcohol outside the home. Previous studies have shown that approximately 30% of parents of teenagers are aware of their child's alcohol use [42,43]. According to an international survey of fifteen year olds from 41 counties, 44% of the adolescents had used alcohol for the first time when they were 13 years old or younger [44]. In a Swedish study of adolescents in the same age range, about 14% of the adolescent reported that they were 13 years old or younger when they were drunk for the first time [45]. It is plausible to conclude that a significant number of the parents in the present study have children who have drunk or tasted alcohol outside the home without their knowledge.

Even if the parents do not perceive their children as susceptible to the specific problem, they do think that underage adolescents should not drink alcohol, at least not outside the home. 92.2% of the parents thought that adolescents should be at least 18 years old before consuming alcohol. This restrictive attitude among Swedish parents has been demonstrated in other studies [46,47]. On the other hand, 34.4% of the parents in the present study had allowed their child to take at least a sip of alcohol from somebody else's glass at home. According to the HBM the target group needs to believe that they can be susceptible to a specific problem and to perceive the problem as serious. Taken together these two components are called perceived threats, and the chance that parents will participate in an intervention increases if they feel a high level of threat [36]. In the present study perceived susceptibility was low while perceived severity was higher. Important is that neither perceived susceptibility nor perceived severity seemed to predict non-participation.

### Program factors: Perceived benefits, perceived barriers and cues to action

Program factors consist of perceived program benefits and barriers together with cues to action. The results showed that perceived program barriers and cues to action seemed to be more important than perceived benefits in predicting non-participation. This result is not compatible with a previous study of parents' inclination to enrol in a parental skills program [37]. That study showed that perceived program benefits were the single most important factor for enrolment. Inclination to enrol in a program has been shown to predict actual participation [15,16]. In the present study one of the items used to measure program benefits tapped parents' perception of the degree to which they held values in common with other parents about the prevention of underage drinking. The reason for this was that shared values among parents in a class were one of the tools used in the current parental program to help parents maintain restrictive attitudes toward adolescents and alcohol. However, this item did not predict non-participation. No significant differences were found in levels of participation between parents who felt that it would be beneficial to have values in common with other parents and those who did not.

The two largest categories from the content analysis, absence of perceived needs and practical barriers, were used in the logistic regression analysis to measure the importance of perceived program barriers. Parents who did not perceive any need for the intervention were more likely to be both partial non-participants and non-participants, while parents who experienced practical barriers were more likely to be partial non-participants. These conclusions seem logical, as parents may want or plan to participate, but because of practical barriers, like time demands, find it difficult to do so.

### Reasons for non-participation: Perceived barriers

Parents reported a wide range of factors that influenced their decision to not participate. Family-related factors seemed to be very important, and not perceiving any need for the program was frequently mentioned. One fifth of the parents reported the latter as a reason for non-participation. Another family-related factor concerned practical barriers which might be possible to alleviate to some extent. For example, it is common that child-care for younger children is provided during parental meetings in parental programs implemented in the US. In this study, time demands were the most prevalent of practical issues. This corresponds with earlier studies [19,26,30]. It is a major challenge for prevention workers to overcome those barriers. In the Iowa Strengthening Families Program, 49% of the eligible families participated after an intensive recruitment procedure, which for example included flyers, letters followed by phone-calls to each family and an economic incentive ($25) [6]. Such an intensive procedure was not possible for the NGO implementing Strong and Clear, because they have limited economical resources. Moreover, in Sweden people are never paid for participating in prevention programs. A Swedish study has shown that the level of interest in parental support through the Internet has increased in recent years [23]. Maybe the Internet is one way to reach parents with important interventions. More studies about what kinds of interventions attract parents and how to increase recruiting and retention of parents in programs and evaluation studies are sorely needed.

### Strengths and limitations of the study

In the present study, a mixed-method design has been used. By combining quantitative and qualitative data, a better understanding of the problem addressed in the study can be provided and a more complete picture can emerge. Furthermore, the combination of quantitative and qualitative data gives a more in-depth knowledge of the participants' perspectives [38]. The combination of the parents' quantitative answers from the three surveys and their written answers about reasons for non-participation provided additional knowledge about non-participation.

Two levels of non-participation were defined in this study, partial non-participation and non-participation. Parents who were designated as partial non-participants had taken part in some activities in the program but they did not consider themselves participants. This is an important distinction, because the parents were allowed to define themselves as non-participants even if they took part in some activities. Further analysis will show if this distinction is important for the effects of the program.

The HBM has been widely used to examine participation in different health interventions, but the use of the model to understand non-participation in parental support programs to prevent underage drinking is limited. This study shows that the model is useful in these kinds of studies as well. It is important, however, to emphasize that the HBM was used as a tool in the analysis and that the main purpose was to understand non-participation in a parental program, not to evaluate the application of the HBM to parental interventions. The model was not used as a guide in planning the study. Nevertheless, many items in the questionnaire fit the major elements in the model theoretically and empirically. A limitation of the study is that perceived barriers were measured by only one item. Moreover, the HBM has been developed over the course of decades and the present study includes the original elements of the model. Another important element, not included in the present study, has since been added to the model, *Self-efficacy *[48]. Self-efficacy is the person's own beliefs about his or her ability to take action [49,50].

It is important to point out that the results of the present study cannot be generalized to the whole Swedish population. The study was carried out in a limited part of Sweden and the selection of schools was not random. For example, there were few parents born outside Sweden. Some of the other socio-demographic factors also had skewed distributions. The majority of the responding parents in the present study were mothers. This is similar to previous research on parental programs [17,19,37]. The dropout rate was higher among parents with a low education level. 53.4% of the parents in the analytical sample had higher education. This can be compared with adults in general in the three municipalities included in the present study aged 29-67 in 2004. 36% had a higher educational level than secondary school [51]. Despite these limitations the present study contributes important knowledge to the understanding of non-participation in a parental program in Sweden.

## Conclusion

To design a parental program that attracts parents independently of their educational level seems to be an important challenge for the future as well as program marketing. From a public health perspective, it is important to promote equity and social justice. Therefore, the recruiting of all kinds of parents is of utmost importance. A main reason for non-participation in a parental program seemed to be absence of a perceived need. However, it is a real dilemma that parents may not always have a valid knowledge about the alcohol consumption among their adolescents. Therefore the program may need innovative measures for raising the awareness among the parents. Moreover, the parents can be more involved in program planning and implementation, which is a measure to empower the parents. This has been successfully implemented in the original Norwegian program Strong and Clear. Practical issues, like time demands, are also important for the level of participation. Therefore, parental support programs could be implemented in other non-school settings such as sport clubs where parents often spend time waiting for their children. To use the Internet is another possibility.

Successful prevention programs need evidence-based methods, but of equal importance is that these programs are attractive and achieve good recruitment as well as retention of parents. Programs are needed which are both high in efficacy and effectiveness in order to have positive population impact.

## Competing interests

The authors declare that they have no competing interests.

## Authors' contributions

CP was the main author of the manuscript and was involved in all aspects of the study. MLB and CE provided scientific oversight and feedback throughout the development of the study and the manuscript. All authors read and approved the final manuscript.

## Pre-publication history

The pre-publication history for this paper can be accessed here:

http://www.biomedcentral.com/1471-2458/9/478/prepub
